# The clinical efficacy and safety of the Chinese herbal medicine Astragalus (Huangqi) preparation for the treatment of acute myocardial infarction: A systematic review of randomized controlled trials

**DOI:** 10.1097/MD.0000000000015256

**Published:** 2019-04-19

**Authors:** Yuqin Zhang, Jiani Wu, Shuwen Guo, Wangou Lin, Binyue Zhang, Xi Chen, Hanrong Mo, Tianwei Zhan

**Affiliations:** a School of Chinese Medicine; b Fangshan Hospital, Beijing University of Chinese Medicine, Beijing, China.

**Keywords:** acute myocardial infarction, astragalus (Huangqi) preparation, protocol, systematic review, traditional Chinese medicine

## Abstract

**Background:**

In recent years, with the enormous advances in the field of cardiac intervention technology, the survival rate of patients with acute myocardial infarction (AMI) has been improved significantly. However, the risk of arrhythmias and heart failure remains very high in AMI patients for long-term prognosis. Chinese herbal medicine (CHM) is more and more used in the treatment of AMI because of its good curative effect and less side effects. The target of this research is to analyze the efficacy and safety of Astragalus (Huangqi) preparation in the treatment of AMI by meta-analysis and also to provide a better evidence for clinical practice.

**Methods:**

Seven databases will be searched in this study: The Cochrane Library, PubMed, Web of Science, the Chinese National Knowledge Infrastructure (CNKI), the Chinese Scientific Journal Database (CSJD), the Chinese Biomedical Literature Database (CBM), and Wanfang DATA. The following search terms will be used: (Huangqi OR Huang Qi OR Astragalus OR radix astragali) AND (acute myocardial infaction OR myocardial infaction OR AMI) AND (randomized controlled trial OR RCT OR randomized). No language limitations and the searches will be conducted up to March, 2019. Inclusion criteria: randomized controlled trial (RCT) of Astragalus (Huangqi) preparation in patients with AMI. Main outcome measures will be left ventricular end systolic volume (LVESV), left ventricular end diastolic volume (LVEDV), left ventricular ejection fraction (LVEF), left ventricular mass index (LVMI), recanalization rate, mortality rate, incidence of reperfusion arrhythmias, postinfarction angina pectoris, and re-infarction rate. Secondary outcome indicators were the incidence of adverse reactions and the effective rate of traditional Chinese medicine (TCM) treatment. Two independent reviewers will filter the literature and extract data which based to the Cochrane manual. The relevant data, including bias risk assessment, data synthesis, subgroup analysis, meta-analysis, and final meta-analysis, will be analyzed with RevMan 5.3 software. The funnel diagram will be used to evaluate the reported deviation, and the Egger test will be used to evaluate the symmetry of the funnel graph.

**Results:**

This systematic review study will provide a clear basis for evaluating the efficacy and safety of Astragalus (Huangqi) preparation with the treatment of AMI.

**Conclusion:**

This study will provide an up-to-date evidence for evaluating the efficacy and safety of Astragalus (Huangqi) preparation.

**PROSPERO registration number:**

CRD42019124843.

## 1 Introduction

Acute myocardial infarction (AMI) is caused by coronary artery occlusion on the basis of coronary artery disease. The blood supply is sharply reduced or interrupted, so that the corresponding acute myocardial ischemia, leading to myocardial necrosis. AMI is a common clinical emergency and critical illness. In recent years, with the development of cardiac intervention technology, the survival rate of patients with AMI has been significantly improved.^[1]^ The treatment of AMI in modern medicine mainly includes drug therapy and percutaneous coronary intervention (PCI), coronary artery bypass graft (CABG). The key is the early rapid opening and reperfusion therapy of infarct-related coronary artery. CABG has a better effect.^[2]^ However, the risk of arrhythmias and heart failure remains very high in patients with AMI remains very high for long-term prognosis.^[3]^ Considerable progress has been made in the treatment of AMI, but mainly by Western medicine and which has obvious toxic and side effects correspondingly.^[4]^

TCM is the crystallization of Chinese wisdom, which plays a vital part in preventing and curing AMI. Astragalus (Huangqi) was contained in the “Shen Nong's herbal classic.” It is often used in a variety of Chinese herbal preparations, can treat many kinds of physical diseases and disorders effectively. The theory of Qi and Blood is an important part in TCM, only the heart-Qi is abundant, the heart rate and rhythm can be maintained, the blood can operate normally in the pulse, and the circulation is constant, nourishing the whole body. Blood stasis is caused by Qi deficiency, the residual blood in the ventricular cavity can not be discharged, cardiac diastolic and contractile function is impaired, causing “real heart pain,” that is, means AMI. Therefore, Qi deficiency and blood stasis is the main pathological mechanism of ischemic heart disease. Tonifying Qi is the basic principle in treating Qi deficiency and Blood stasis. Astragalus (Huangqi) preparation as a representative drug for the treatment of Qi deficiency syndrome, contains astragalus polysaccharides, astragalus saponins, and other components. At present, it is mainly used in immunomodulation, antioxidation, anti-inflammatory, and anticancer effects. In cardiovascular studies, Astragalus (Huangqi) preparation has a protective effect on the heart, including promoting cardiac microvessel formation,^[5]^ improving cardiac myocyte hypertrophy,^[6,7]^ reducing myocardial apoptosis,^[8]^ reducing inflammatory factor injury,^[9]^ relieving excessive oxidative stress and improving energy metabolism disorder.^[10]^ There are many clinical reports about Astragalus (Huangqi) preparation in the treatment of AMI, but the sample size of these studies is small, the evidence level is not high, and there is no systematic analysis to assess the efficacy and safety of Astragalus (Huangqi) preparation in the treatment of AMI. Therefore, the key of this study is to analyze the efficacy and safety of Astragalus (Huangqi) preparation in the treatment of AMI by meta-analysis and also to provide a better evidence for clinical practice.

## 2 Method and analysis

### 2.1 This research program has been registered in the PROSPERO, and PROSPERO registration number is: CRD42019124843

The procedures for this protocol will be put into effect in the light of the priority reporting projects of the PRISMA-P guidelines.

### 2.2 Inclusion criteria for study selection

#### 2.2.1 Types of studies

We will include all the RCTs that investigated the clinical efficacy and safety of Astragalus(Huangqi) preparation combined with conventional drug for the treatment of acute myocardial infarction. Astragalus (Huangqi) preparation can be used in a variety of forms, such as granules, injections, decoction etc. We will not accept the use of Astragalus (Huangqi) combined with other Chinese herbal Medicine. We will exclude trials using quasi-random methods. Without restrictions on blinding, language, and publication time. Letters, comments, case reports, and case series will be excluded.

#### 2.2.2 Types of patients

The objects of the study will be patients clinically diagnosed with AMI, regardless of their age, sex, race, level of education, financial status, and whether they are outpatients or in-patients. The diagnostic criteria for AMI should be based on one of the past or present definitions.

#### 2.2.3 Outcome measures

The primary outcome measures will be left ventricular end diastolic volume (LVEDV), left ventricular end systolic volume (LVESV), left ventricular mass index (LVMI), left ventricular ejection fraction (LVEF), recanalization rate, mortality rate, incidence of reperfusion arrhythmias, postinfarction angina pectoris, and re-infarction rate.

The secondary outcome indicators will be the incidence of adverse reactions and the effective rate of TCM treatment.

### 2.3 Search methods for the identification of studies

#### 2.3.1 Electronic search

Seven databases will be searched in our research: The Cochrane Library, Web of Science, PubMed, CBM, CNKI, CSJD, and Wanfang DATA. No language limitations and the searches will be conducted up to March, 2019.

#### 2.3.2 Searching other resources

At the same time, we will search for relevant journals in references and track them to avoid omitting any related research, and use Internet search engines, such as Google Scholars, to detect related documents manually. Meanwhile, we will search for completed but unpublished clinical trials and track the findings.

#### 2.3.3 Search strategy

The specific search strategy used will be as follows (taking PubMed as an example):

#1 “acute myocardial infarction”[Title/MeSH] OR “myocardial infarction”[Title/MeSH]
#2 “efficacy”[Title/Abstract] OR “safty”[Title/Abstract] OR “efficacy and safty”[Title/Abstract]
#3 “Huangqi”[Title/MeSH] OR “Huang Qi”[MeSH] OR “Astragalus”[Title/MeSH] OR “radix astragali”[MeSH]
#4 “randomized controlled trial”[MeSH] OR “RCT”[MeSH] OR “randomized”[MeSH]
#1 AND #3 OR #2 OR #4

### 2.4 Data collection and analysis

#### 2.4.1 Selection of studies

**Figure 1:**
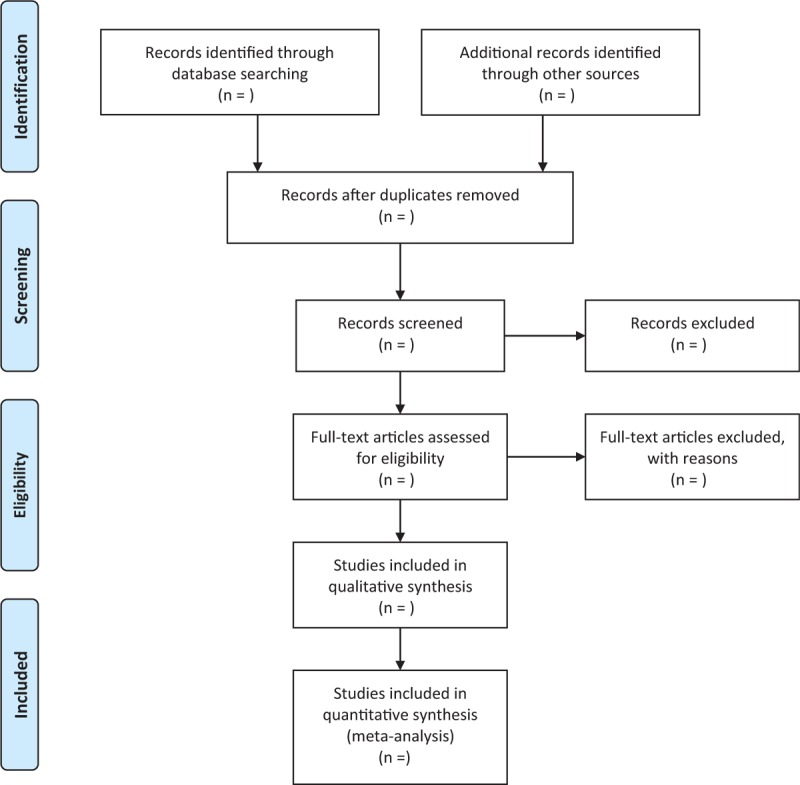
Flow diagram of study selection process.

The authors (ZY and WJ) will extract and check data to make independent evaluations to find possible eligible studies. Articles in this study that are repeated or do not meet the eligibility criteria, interventions, or outcomes will be excluded. We will extract data from articles which compliance with inclusion criteria. Differences will be resolved by discussion or arbitration by a third author (GS), if necessary. The following data items will be extracted: first author, year of publication, patient age, gender, diagnostic criteria, sample size, blindness, intervention, research data, binary variable data and continuous variable data in outcome indicators, follow-up time, Shedding cases, adverse events, etc. The procedure of studies Selection is presented in PRISMA flow diagram. (Fig. 1)

#### 2.4.2 Assessment of risk of bias in included studies

The literature will be screened independently by 2 researchers according to the criteria of inclusion and exclusion. On the basis of the risk of bias tool which is supplied by cochrane Collaborative Network, the following aspects of the selected literature will be evaluated: random sequence generation, allocation of concealment, blindness of the subjects and implementers, and blindness of the outcome evaluators. The outcome data is incomplete, selective reporting results, other bias, finally to each item to make high bias risk, low bias risk, bias risk uncertain 3 kinds of evaluation; Evaluation of evidence for inclusion studies using GRADE^[11,12]^ for analysis: bias risk, inconsistency, inaccuracy, publication bias, And make the judgment of “extremely low” or “high” for each grade of evidence. The results were cross-checked and resolved by the third researcher when there were differences.

#### 2.4.3 Measures of treatment effect

The RevMan 5.3 software, which is provided by Cochrane Collaboration Company, will be used to analyze the data. OR, SMD and 95% CI will be used as the effective quantity. Firstly, χ^2^ test (chi-square test) will be used to analyze the statistical heterogeneity and *I*^2^ value was used to estimate the size of heterogeneity. If *P* > .1, *I*^2^≤50% can be considered to be homogeneous among the multiple studies included, and fixed effect model can be chosen if *P*≤. 1. If *I*^2^ > 50% can be considered to be heterogeneous among the multiple studies included, the source of heterogeneity can be confirmed firstly through subgroup analysis or sensitivity analysis, and if the source of heterogeneity cannot be judged or eliminated, random effect model should be chosen. If *P* < 0.05 the difference is statistically significant. The funnel diagram will be used to estimate whether there is publication bias. Egger test will be used to evaluate the symmetry of funnel graph, and the value of *P* < 0.1 will be interpreted as statistically significant.

#### 2.4.4 Dealing with missing data

If there is doubt and lack of raw data, we will contact the author for more information, if not obtained, we will use available data for analysis and discuss the possible impact of missing data on the results.

#### 2.4.5 Assessment of reporting bias

A funnel plot will be used to discuss the reporting bias if more than 10 trials are included in the study.

#### 2.4.6 Sensitivity analysis, subgroup analysis and meta-regression

If heterogeneity is found, sensitivity analysis or subgroup analysis or meta-regression analysis will be used. According to the characteristics of this study, the size of samples, the severity of acute myocardial infarction, the different dosage and dosage of Astragalus membranaceus preparation, the treatment time and other related parameters, will be analyzed to explore the potential sources of heterogeneity.

### 2.5 Ethics and dissemination

This review does not require ethical recognition. The study is based on published data and does not focus on patients’ privacy. The meta-analysis results will be reported in accordance with the PRISMA extension statement and published in peer-reviewed journals.

## 3 Discussion

Huangqi has been used clinically in China for thousands of years. At present, Astragalus (Huangqi) preparation has been widely used in the replacement therapy of AMI patients. More and more clinical studies have shown that Astragalus (Huangqi) preparation has great benefits in improving patients with AMI. However, the clinical evidence of Astragalus (Huangqi) preparation as evidence-based medicine for the intervention of AMI has not been systematically evaluated. Therefore, we intend to conduct this systematic review to evaluate the efficacy and safety of Astragalus (Huangqi) preparation in patients with AMI. It is hoped that this study can provide more convincing evidence to prove the advantage of Astragalus (Huangqi) preparation in AMI and provide reference for clinical application. However, there may be some potential defects in this study. First, different doses of herbal medicine, patient age and the severity of AMI may be at risk of heterogeneity. Finally, small samples can lead to a high bias risk.

## Author contributions

**Conceptualization:** Yuqin Zhang, Shuwen Guo.

**Data curation:** Yuqin Zhang, Jiani Wu.

**Formal analysis:** Wangou Lin, Binyue Zhang.

**Funding acquisition:** Shuwen Guo.

**Investigation:** Yuqin Zhang, Jiani Wu.

**Methodology:** Yuqin Zhang, Shuwen Guo.

**Project administration:** Yuqin Zhang.

**Resources:** Xi Chen, Hanrong Mo, Tianwei Zhan.

**Software:** Wangou Lin, Binyue Zhang.

**Supervision:** Yuqin Zhang, Shuwen Guo.

**Validation:** Shuwen Guo.

**Visualization:** Xi Chen, Hanrong Mo, Tianwei Zhan.

**Writing – review & editing:** Yuqin Zhang, Jiani Wu.

**Writing – original draft:** Yuqin Zhang
